# The characterization of bearded vulture (*Gypaetus barbatus*) coprolites in the archaeological record

**DOI:** 10.1038/s41598-022-25288-x

**Published:** 2023-01-03

**Authors:** Montserrat Sanz, Joan Daura, Ana Maria Costa, Ana Cristina Araújo

**Affiliations:** 1 Grup de Recerca del Quaternari, GRQ-SERP, Department of History and Archaeology, Universitat de Barcelona, 08001 Barcelona, Spain; 2 Faculdade de Letras, UNIARQ-Centro de Arqueologia da Universidade de Lisboa, Universidade de Lisboa, 1600-214 Lisbon, Portugal; 3 Laboratório de Arqueociências (LARC)-DGPC, Calçada do Mirante à Ajuda, nº 10A, 1300-418 Lisbon, Portugal; 4 InBIO Laboratório Associado, BIOPOLIS - Programme in Genomics, Biodiversity and Land Planning, CIBIO - Centro de Investigação em Biodiversidade e Recursos Genéticos, Vairão, Portugal; 5 IDL - Instituto Dom Luiz, Universidade de Lisboa, Edifício C6, Piso 3, 1749-016 Lisbon, Portugal; 6 IIIPC - Instituto Internacional de Investigaciones Prehistóricas de Cantabria, Universidad de Cantabria - Gobierno de Cantabria-Santander, Avda de los Castros 52, 39005 Santander, Spain

**Keywords:** Archaeology, Palaeontology

## Abstract

The archaeological record of the Lagar Velho rock shelter (Lapedo Valley, Leiria, Portugal) bears testimony to several significant Upper Palaeolithic occupations, most notably the *Lapedo Child* burial (LV1) dating from the Gravettian. Excavations undertaken at the site since 2018 have seen the recovery of a large quantity of coprolites, above all in layer 143 (c. 29 ka cal BP). The study of these fossilized remains points to the bearded vulture (*Gypaetus barbatus*) as the main coprogenic agent and provides the first descriptions of these avian coprolites in archaeological assemblages. The analyses reported involved the comparison of the coprogenic samples with modern bearded vulture scats. A new morphotype is proposed for discriminating the faeces of this avian scavenger based on (1) macroscopic analyses, (2) morphometric comparisons with other fossil and modern scats and (3) their mineralogical and elemental composition. Among the criteria proposed here to identify the coprolites of the bearded vulture are their cylindrical shape, diameter, pointed extremities and homogeneous porous texture, as well as their massive internal texture, hard consistency and total absence of bone inclusions (attributable in all likelihood to a high digastric juice acidity capable of dissolving bones). Our results indicate that, as well as being used by humans for short-term stays, the Lagar Velho rock shelter was used by the bearded vulture as a nesting site. We provide new evidence from Iberia of the activity of this avian scavenger as a bone accumulator in archaeological sites.

## Introduction

The bearded vulture (*Gypaetus barbatus*) is an avian scavenger and bone-eater with physiological adaptations and digestive abilities, such as a flexible oesophagus and a low stomach acidity that allows it to swallow and digest macro-mammalian bones of up to 30 cm in length^1,2^. Bearded vultures are known to select the most fatty and nutritive bones in order to maximise the properties of the food and the energy obtained, and to optimise their foraging time^3–5^. It is the only species of animal with a bone-based diet^1,5,6^ (in which bones account for between 70 and 90%), having learnt to drop bones that are too long to swallow on the rocky slopes (bone-breaking sites or ossuaries) of its habitat^7^, acquiring in the process the name of ‘bone breaker’—or *quebra-ossos* in Portuguese, *quebrantahuesos* in Castilian and *trencalòs* in Catalan. The vultures then carry the bones or bone fragments to their nests, which are typically built in the openings and ledges of rugged areas, often limestone cliffs^8^. In these areas there are many caves and rock shelters accessible to humans which could have served as a habitat for Palaeolithic hunter-gatherers. However, fossil remains ascribed to the bearded vulture in the Iberian Pleistocene record are rare, although this avian scavenger has been identified in various Pleistocene sites, including Quibas (Murcia), Gorham’s cave (Gibraltar), Caldeirão cave (Portugal), and Santa Catalina (Bizkaia), among others^9–12^. The negligible representation of this species in the fossil record is most likely related to the scarcity of avifauna studies. Yet, a number of taphonomic studies have identified the bearded vulture as an agent of bone accumulation, for instance, in the cave sites of El Mirón (Spain)^13^, Mavro Mouri (Crete)^14^, Luri-Grítulo (Corsica)^15^, and Noisetier (France)^16^ and, tentatively, in Caldeirão (Portugal)^9^.

The bearded vulture has at least three behavioural traits in common with Palaeolithic humans and hyenas. First, it usually feeds usually on medium-sized ungulates; second, it accumulates bones; and third, it lives in openings and ledges in or above caves and rock shelters. Despite its obvious capacity to accumulate bone assemblages in archaeological sites, little is known at present about its activity. However, Marín-Arroyo and Margalida have proposed certain key features—e.g., bone surface alterations, breakage patterns and skeletal profiles—that should be considered to identify the presence of the bearded vulture in archaeological sites^17^, completing in this way a number of earlier studies, most notably that of Robert and Vigne^15^. Here, it has been pointed out that certain features (in particular, digestive traces left on bones regurgitated by the bearded vulture due to their low gastric pH, below 1)^18^, have often been misinterpreted as resulting from other causes^14^, such as osteoporosis, or attributed to other carnivore agencies, the case of remains in Pego do Diabo cave (Portugal)^19^.

The stomach of the bearded vulture produces almost pure hydrochloric acid, which ensures the bones consumed are fully digested. The bone minerals—primarily phosphorous and calcium^2^—evacuated in the faeces, generate hard, dry whitish pellets, referred to by Spanish biologists as *tizas* (in Castilian) because of their resemblance to a stick of blackboard chalk. Research at the Noisetier cave indicated the possible presence of bearded vulture coprolites for the first time in an archaeological record^16^. However, these fossil faeces were fragmentary and were not described in any detail.

Scats contain useful information for biologists, allowing them to monitor animal populations and assess their health, diet, reproduction, and other factors using non-invasive techniques. However, coprolites found in archaeological assemblages have attracted relatively little attention. Hyena coprolites are the most frequently identified fossilized faeces in the archaeological record, thanks to their distinctive shape and good preservation^20,21^. In recent decades, interest in determining other non-hyenid coprolites in assemblages has grown, based essentially on morphological and morphometric criteria, which are helpful in distinguishing different predator sizes^22–24^.

The aim of this study is to identify, describe in detail, and characterise for the first time ever the archaeological coprolites of the bearded vulture (*Gypaetus barbatus*) in samples collected from the Lagar Velho rock shelter (Leiria, Portugal), dated to c. 29 ka cal BP. Our identification of bearded vulture coprolites relies mainly on morphological, morphometric, and compositional analyses performed on modern-day bearded vulture scats collected in the Aragonese Pyrenees (Huesca, Spain), from the Ordesa and Monte Perdido National Park populations, and on the scats of chicks held at the Breeding Centre for the Lammergeier in Human Isolation (CRIAH) located in Pastriz (Zaragoza, Spain).

## Background

**Figure 1:**
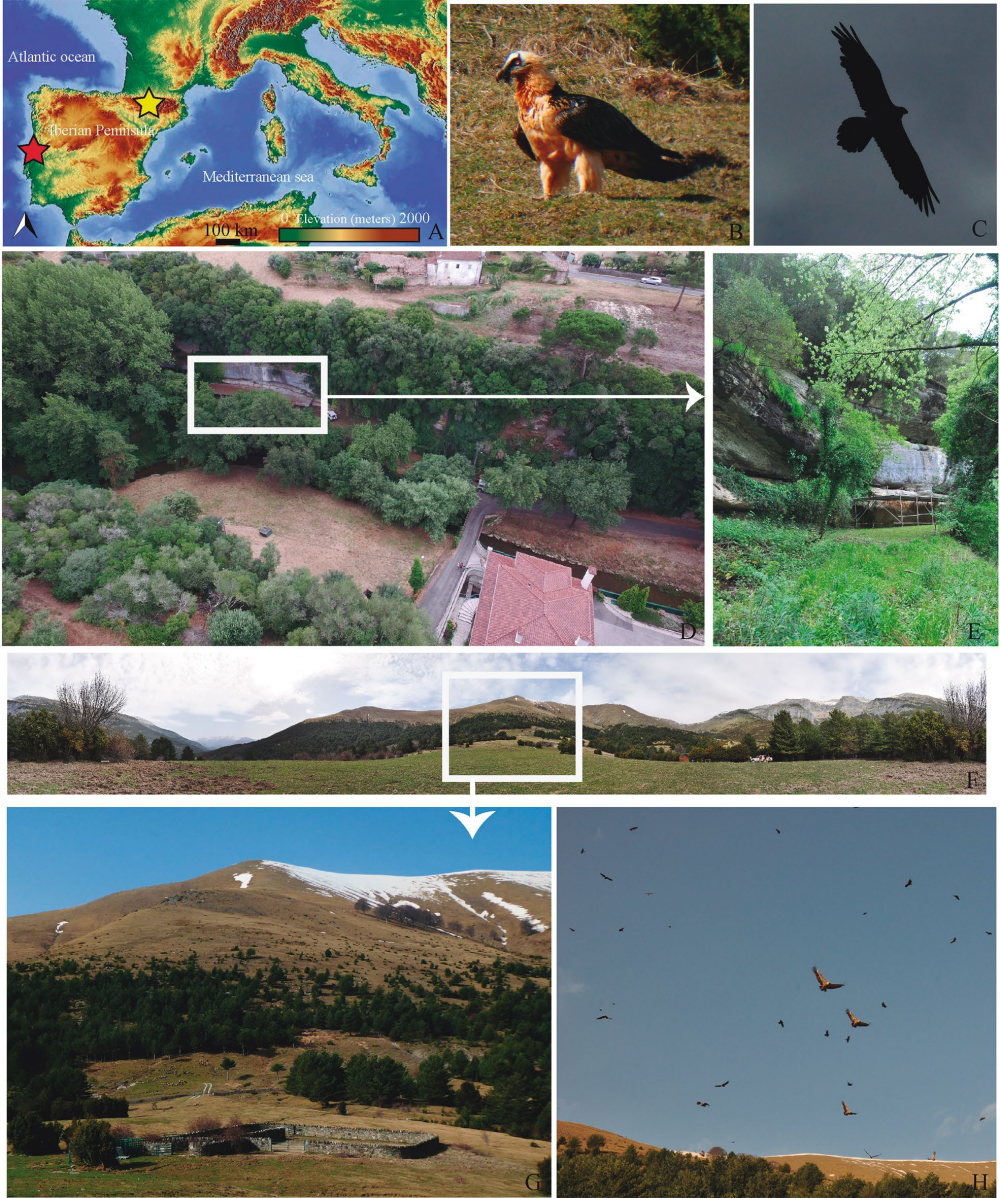
(**A)** Map showing the location of the Lagar Velho rock shelter (red star) in Portugal and the Ordesa and Monte Perdido National Park (yellow star) in Spain. Map extracted from OpenStreetMap (CC BY-SA). OpenStreetMap© licensed under ODdL 1.0 (https://www.openstreetmap.org/copyright) by the OpenStreetMap Foundation (OSMF). ©OpenStreetMap contributors (https://www.openstreetmap.org/). The licence terms can be found on the following link: http://creativecommons.org/licenses/by-sa/2.0/ (accessed on 21 May 2022). (**B**) and (**C**) Bearded vultures in the supplementary feeding station located in the Ordesa and Monte Perdido National Park (PNOMP). (**D**) and (**E**) Lagar Velho rock shelter (D: Aerial view of the shelter). (**F**) and (**G**) General view of the supplementary feeding station located in the PNOMP where current scats of bearded vulture were collected. (**H**) Vultures (mostly griffon, but also bearded and modern Egyptian vultures) circling over the feeding station (H).

Lagar Velho (henceforth, ALV) is a rock shelter located in the Lapedo Valley (30°45′25″N; 8°43′58″W), near the mouth of the Lapedo gorge, on the left bank of the Caranguejeira stream. The site is about 135 km north of Lisbon and lies in the municipality of Leiria (Fig. 1). Hunter-gatherer groups took advantage of the site’s optimal geographical and geomorphological setting, which is formed by an elongated platform of ~ 125 m^2^, facing north, and its watercourse. Lagar Velho was discovered and first excavated in 1998, soon after the identification of the LV1 child burial^25,26^.

Upper Palaeolithic occupations dated to the Middle Solutrean (c. 24 ka cal BP), Proto-Solutrean (c. 26 ka cal BP) and the Gravettian (c. 27–30 ka cal BP) have been identified in different sectors of the shelter^26,27^. The sedimentary succession is complex, presenting great vertical and lateral variability^27^ as reflected by its stratigraphy, pedosedimentary components and archaeological record. However, the top sedimentation was mainly dependent on gravity-driven processes, while fluvial sediments are largely present at the base of the sequence.

The coprolites analysed in this paper were recovered from layer 143, the stratigraphic unit targeted during the recent excavations at the site that started in 2018 (SI Fig. 1). Layer 143 is located in the western area of the site and is dated c. 29 ka cal BP. No direct correlation between this layer and the LV1 child burial (located in the eastern part of the site) has yet been described, but the new archaeological studies are shedding new light on the relation between the eastern and western areas of the site. Different units presenting human input related to fire activity have been identified in the same stratigraphic complex of layer 143. Although zooarchaeological and taphonomic studies of layer 143 are still in progress, the large mammals recovered are clearly dominated by red deer, followed by large bovids, wild boar, roe deer and equids. Carnivore remains are scarce in this layer. The assemblage is heavily thermoaltered, showing different degrees of burning ranging from slightly burned to fully calcined (completely white in colour), with some fragments showing refitting in situ due to the action of the fire. Preliminary observations^28^ identified modifications produced by carnivores (pits, punctures, scores and chewing portions) and a few striations that might correspond to anthropogenic marks. Fresh fractures in bones are the most abundant, due to the extraction of marrow from long bones by biological agents, but dry fractures due to fire action are also present. Layer 143 is located immediately below the EE15 occupation surface, described as a red deer hide processing camp^27^. Nevertheless, zooarchaeological results point to the involvement of more complex taphonomic processes and biological agents in the accumulation and modification of layer 143, thus opening up new perspectives on the interpretation of this Gravettian unit.

**Figure 2:**
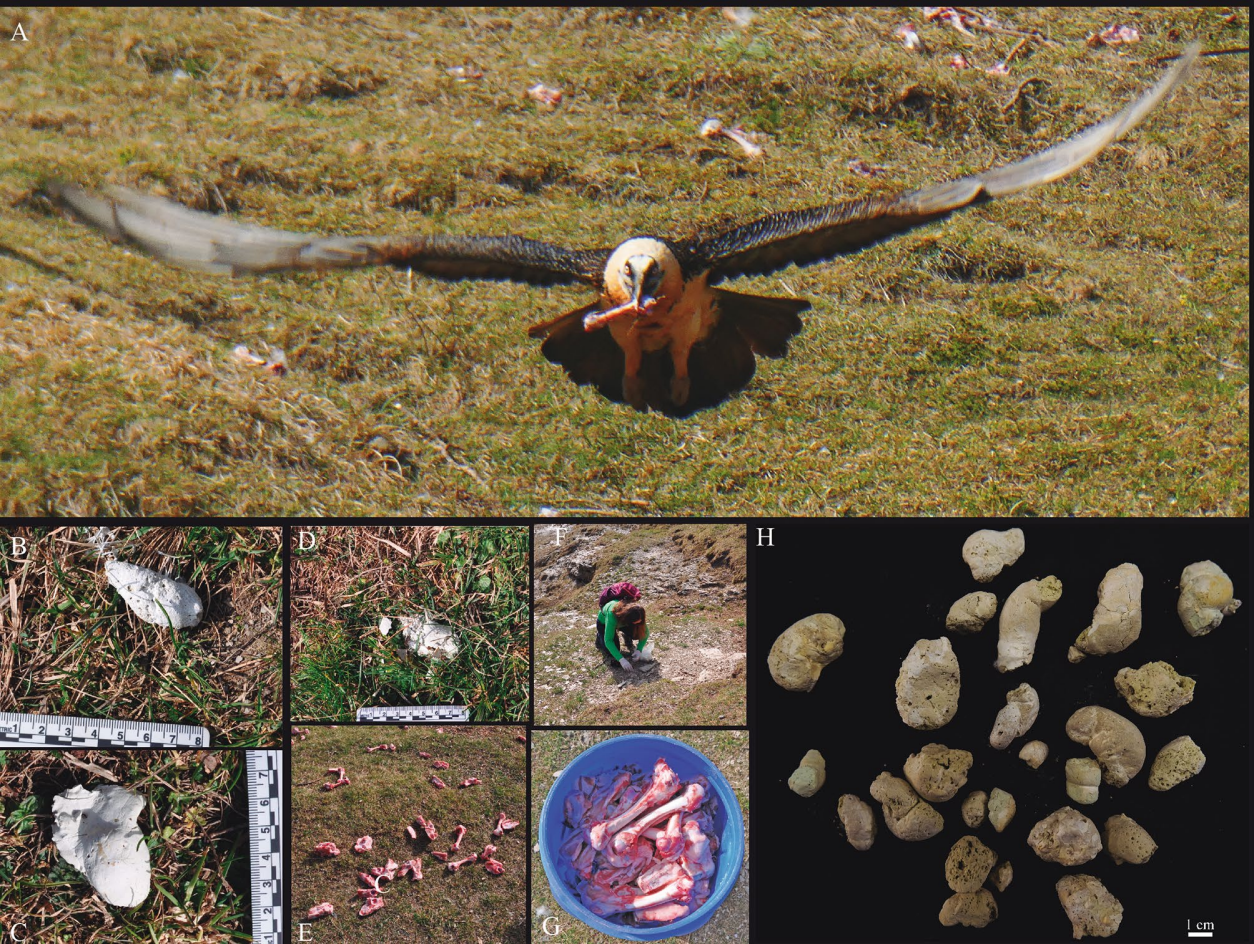
Supplementary feeding station located in the Ordesa and Monte Perdido National Park. (**A**) Adult bearded vulture with a sheep bone while flying. (**B**,**C**) and (**D**). Bearded vulture scats recovered for the present study. (**E**) and (**G**) Sheep bones used to feed vultures. (**F**) One of the authors collecting faecal samples. (**H**) Adult bearded vulture scats recovered from a modern-day nest (Huesca, Spain).

Moreover, preliminary analyses carried out by one of the authors (M.S.) on the faunal material recovered during the 2018 archaeological campaign led to the identification of ungulate bones that presented extreme physical and biochemical alterations, including shiny surfaces, cortical bone slimming and several perforations caused by chemical agents. These diagnostic features point to dissolution in a high acidity (low pH) environment, such as the digestive tract of bearded vultures. If we then consider these features in combination with the anatomical parts affected^15,17^, notably phalanges, it seems clear that we are dealing with the bearded vulture (SI Fig. 2). To corroborate the hypothesis that the site was occupied by this species, together with the coprolites, we analysed modern bearded vulture faeces collected by biologists and other professionals working in the field of vulture conservation. These samples were gathered from nests located in serveral areas of Aragón (Spain) and from feeding stations located in the Ordesa and Monte Perdido National Park (Spain) (Figs. 1, 2). Scats from chicks (excreted between 40 and 100 days after birth) were also considered in this study and were provided by *Fundación para la Conservación del Quebrantahuesos* (FCQ) from the Breeding Centre for the Lammergeier in Human Isolation (CRIAH).

## Results

### Macroscopic analyses

The coprogenic assemblage from ALV and the modern bearded vulture faeces were both examined macroscopically, focusing on their preservation, external morphology, the shape of their extremities, internal texture, hardness and constriction lines.

**Figure 3:**
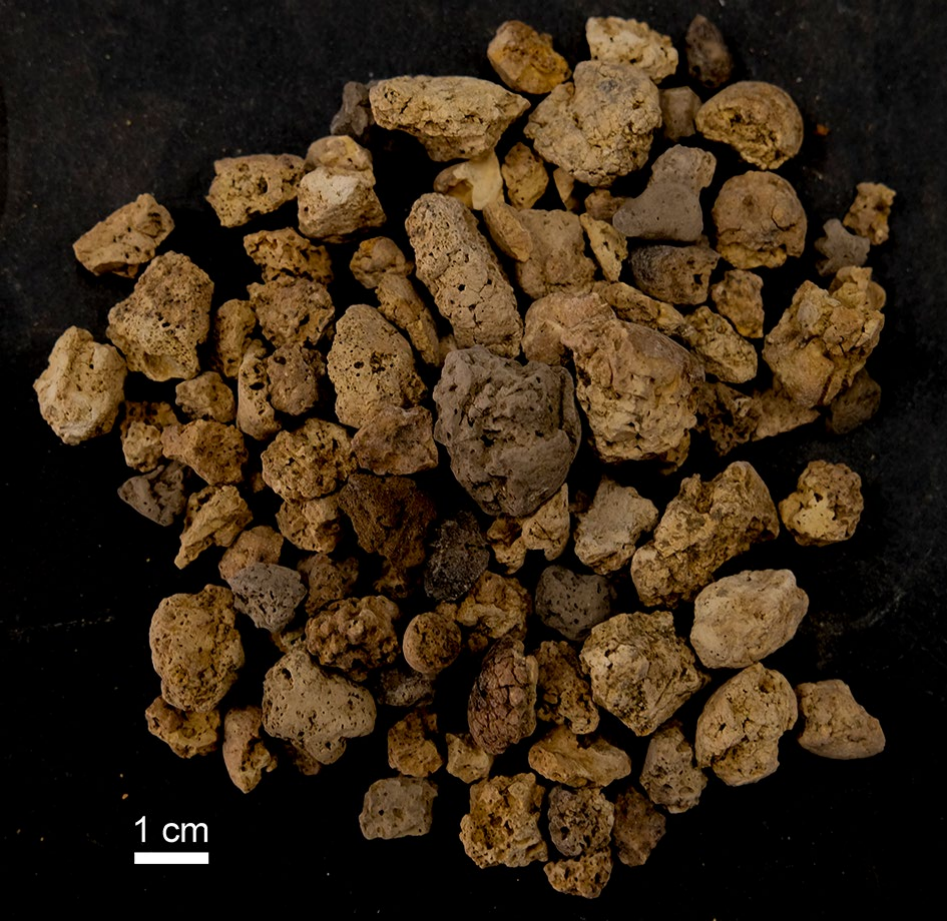
Shapeless coprolites recovered after sieving layer 143 sediments (square G6). Some specimens are burned due to human activity at the site.

**Figure 4:**
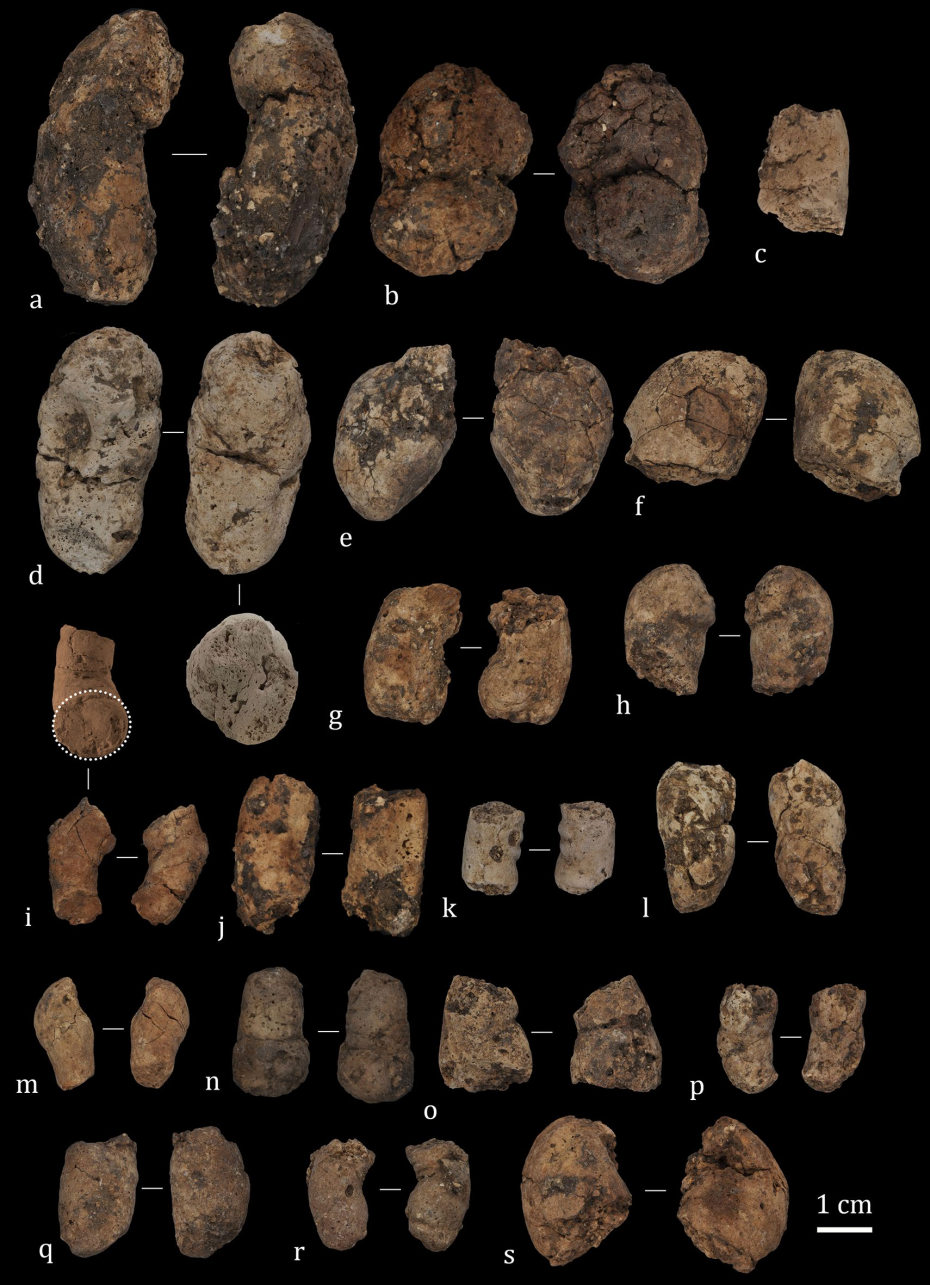
Coprolites recovered from ALV layer 143, highlighting their internal texture (white discontinuous circle) (**i**). Compositional characterization was performed on coprolites (**c)** (sample #3) and (**d**) (sample #2).

The ALV coprogenic collection is dominated by shapeless specimens (Fig. 3) and for this study, we selected fragmentary (N = 72) and complete (N = 4) specimens (Fig. 4). The modern bearded vulture scats are both complete (N = 10 adults and N = 37 chicks) and fragmentary (N = 26 adults and N = 31 chicks).

The ALV coprolites present, in the main, a cylindrical morphology (N = 74) and a porous internal texture (N = 60). Their extremities are poorly preserved (N = 121), exhibiting pointed (N = 10), rounded (N = 19) and, occasionally, flat (N = 2) ends. A hard consistency is observed in all the ALV samples. Single pellets are the most abundant, with little evidence of segmentation with one (N = 3) or two constriction lines (N = 2). The external surfaces and sections of the fragmented coprolites were examined for inclusions and bone content. No bones or other inclusions were observed on the external surfaces of ALV coprolites. As shown in Fig. 4i the internal texture is porous and without inclusions.

Coprolites from ALV are mineralized and the original shape is preserved in many specimens. Moreover, few disaggregated coprolites were found at the site, indicating low atmospheric exposure before burial. By contrast, the high degree of breakage and the poorly preserved extremities may be the result of trampling, sediment pressure and the activity of other biological agents. ALV coprolites present various colours, ranging from a yellowish-brown to whitish-grey.

**Figure 5:**
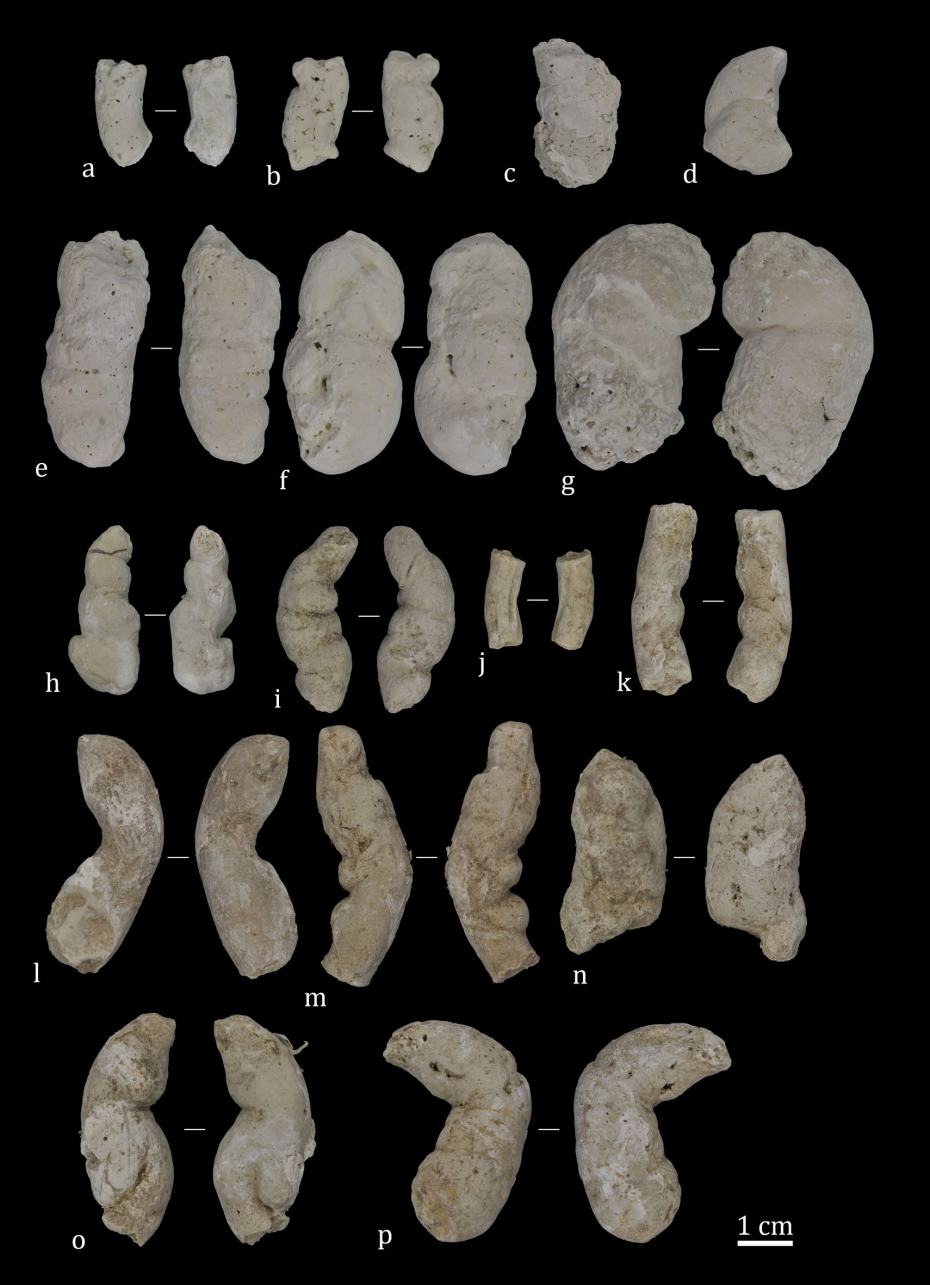
Modern bearded vulture scats. (**a**) to (**g**). Adult scats recovered from nests in the Aragonese Pyrenees (Huesca, Spain). (**h**) to (**p**) Chick (between 40 to 100 days of life) scats from the Breeding Centre for the Lammergeier in Human Isolation (CRIAH) (Zaragoza, Spain).

As a comparison, a collection of modern-day bearded vulture scats was studied (Fig. 5). These modern-day scats have a cylindrical shape (N = 100), with three specimens presenting a globular morphology. They are better preserved than the ALV coprolites and show round (N = 44) and pointed (N = 53) extremities, with only a few presenting flat extremities (N = 3). Their internal texture is porous (N = 96) and massive (N = 7), and all present a hard consistency. Single pellets are the most abundant in the assemblage, with little evidence of segmentation, and the occasional presentation of one (N = 11), two (N = 6) and three (N = 15) constriction lines. No bones were observed in the external surfaces of bearded vulture faeces. Modern-day bearded vulture scats are greyish-white in colour.

**Figure 6:**
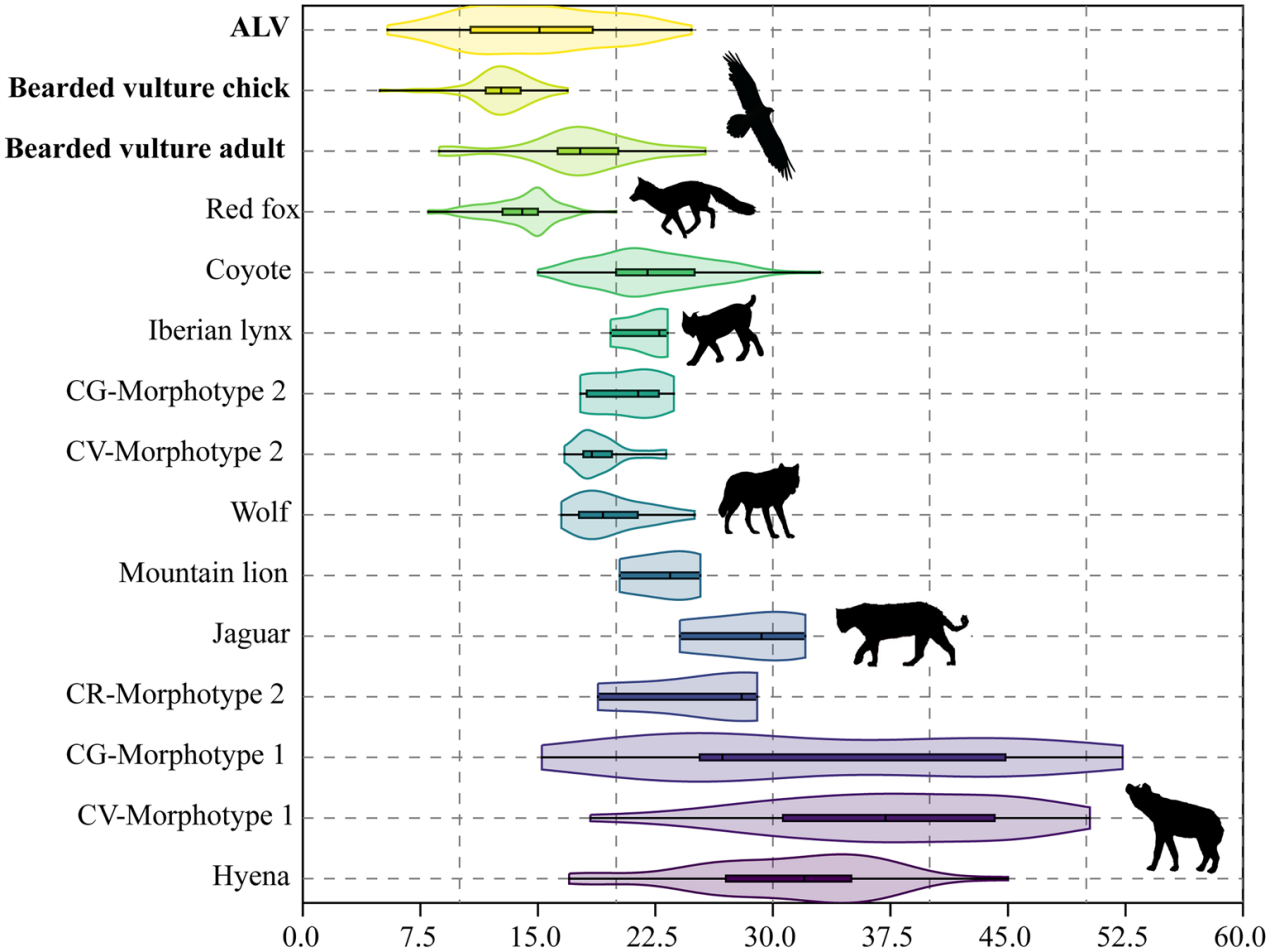
Violin plot of the diameter of bearded vulture scats and ALV coprolites (present study) compared to modern-day scats and coprolites according to morphotype (in mm)^22,23,30–32^. Morphotypes 1 and 2 refer to hyenid and non-hyenid carnivores, respectively. CG: Cova del Gegant. CV: Cova del Coll Verdaguer. CR: Cova del Rinoceront.

Maximum length and diameter measurements of well-preserved coprolites are a valuable tool for discriminating agents. In Fig. 6, the diameters of ALV coprolites and bearded vulture faeces (Supplementary Table 1) are plotted together with those of the coprolites and modern-day scats of other agents^22,23,29–32^. As can be seen, the dimensions of the ALV coprolites (mean diameter of 14.9 mm, ranging from 5.4 to 24.8 mm) clearly coincide with the range presented by adult bearded vulture scats (*Gypaetus barbatus*, mean diameter of 17.4 mm) and chick scats (*Gypaetus barbatus*, mean diameter of 12.7 mm) (Table 1), but also with that of red fox faeces (*Vulpes vulpes*, mean diameter of 14 mm)^33^. The ALV coprolites are clearly distinct from those of hyena (*Crocuta crocuta*), based on their smaller diameter (ranging between 5.4 and 24.8 mm), but also from other non-hyenid coprolites and scats, including the Iberian lynx (*Lynx pardinus*, mean diameter of 21.8 mm), wolf (*Canis lupus lupus*, mean diameter of 19.7 mm) and large felids, such as mountain lion (*Puma concolor*) and jaguar (*Panthera onca*).

**Table 1:** Measures (maximum length and diameter, in mm) of bearded vulture scats (chicks and adults) and ALV coprolites.

	Bearded vulture chick		Bearded vulture adult		ALV	
	Length (mm)	Diameter (mm)	Length (mm)	Diameter (mm)	Length (mm)	Diameter (mm)
N	65	65	31	31	71	71
min	12.5	4.9	10.3	8.7	6.5	5.4
max	46.7	16.9	51.7	24.3	51.2	24.8
Mean	29	12.7	29.3	17.4	22	14.9
σ	6.3	2.1	9.2	3.7	8.6	4.6

### Chemical analyses

Four ALV coprolites (samples #1 to #4) and five bearded vulture faeces (samples #5 to #9) were selected for compositional characterization (Supplementary Table 2). Two ALV samples are illustrated in Fig. 4: sample #2 correspond to d and sample #3 to c.

**Figure 7:**
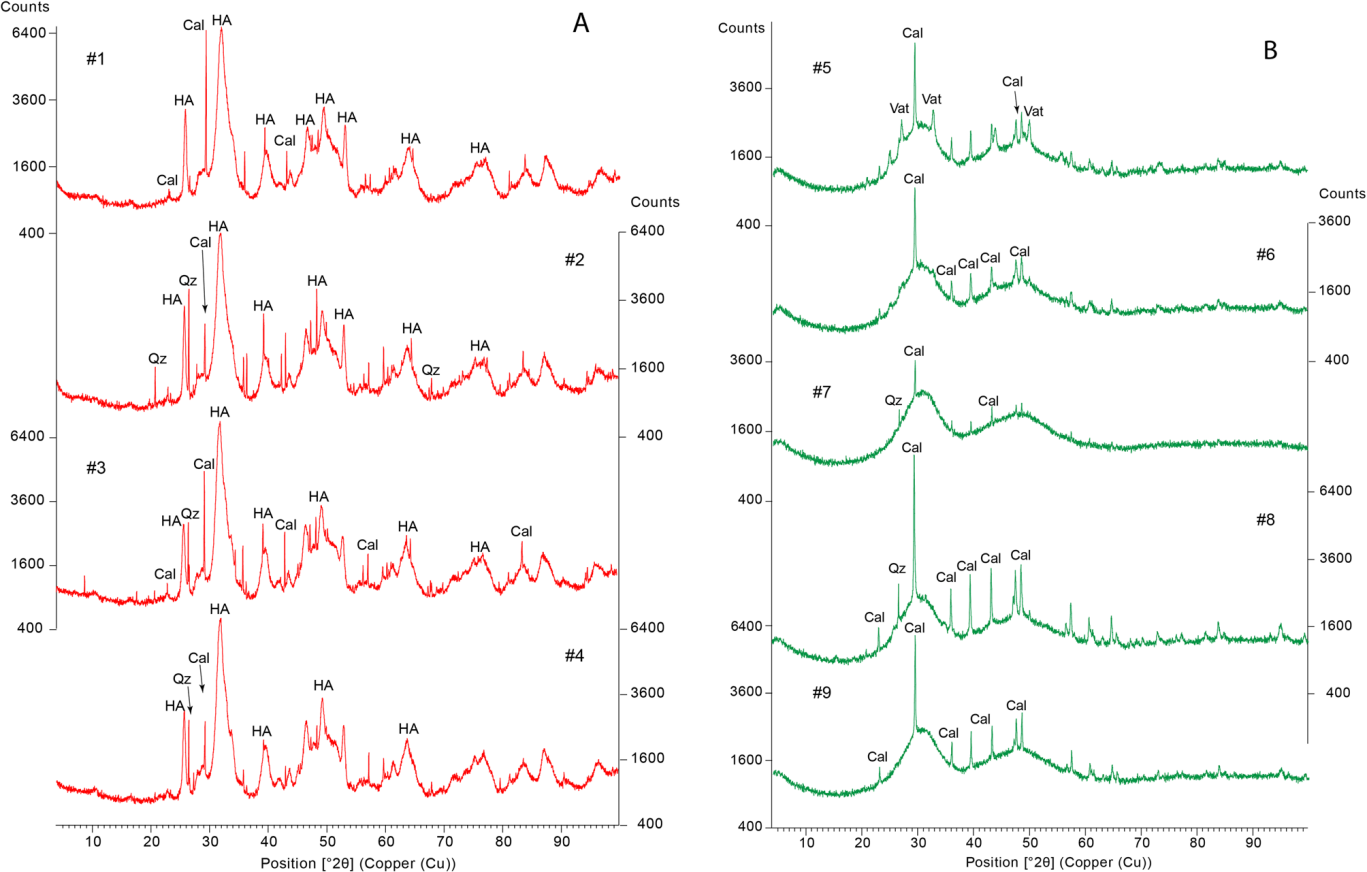
Mineralogy results using X-ray diffraction (XRD). (**A**)—Coprolites recovered at ALV archaeological site. (**B**)—Modern bearded vulture scats. HA—hydroxyapatite; Cal—calcite; Qz—quartz; Vat—vaterite.

The X-ray diffraction (XRD) results for the coprolites and the modern scats are shown in Figs. 7A,B, respectively. The four coprolite ALV samples are similar, being found to contain essentially hydroxyapatite (Ca_5_(PO_4_)_3_OH; HA), a calcium phosphate complex resulting from the digestion of bone in the bearded vulture gastrointestinal tract^2^. The hydroxyapatite peaks are broad, reflecting poor mineral crystallization. In addition, all the XRD diffractograms present sharp peaks associated with calcite (CaCO_3_), the highest intensity being recorded in samples #1 and #3. Quartz (SiO_2_) is also present in all coprolites, with the exception of sample #1. Both calcite and quartz are present in the Lapedo Valley, being associated with karstic systems (mostly limestone disaggregation) and fluvial sands. The presence of calcite may also result from the recrystallization of calcium carbonate after the dissolution of bones.

The results for the five modern scat specimens (Fig. 7B) are very similar, but differ from those of the coprolite specimens. Results point to a greater number of amorphous phases, which may be related to the presence of organic materials or amorphous calcium phosphates^34^, precluding accurate XRD analyses. Notwithstanding, sharp peaks of calcite were identified in all five diffractograms, with the highest intensities being recorded in samples #8 and #9. Quartz (SiO_2_) was only identified in samples #7 and #8 and in sample #5 peaks associated with vaterite (CaCO_3_) were also identified. Vaterite is a highly unstable polymorph of calcium carbonate that frequently reverts to other more stable forms such as calcite. However, vaterite crystals seem to be produced by some species of the genus Saxifraga^35^, alpine plants that may also occur in the Pyrenees, in the area from which modern scat samples were collected. The presence of vaterite in the modern scats also seems to indicate that these faeces were defecated very shortly before being collected.

As the amorphous composition of modern faeces prevents our obtaining accurate XRD results, elemental analyses of calcium (Ca) and phosphorus (P) were also performed in modern-day samples. All samples present a calcium content higher than 25% and a phosphorus content ranging from 11.4 to 13.4% of total fecal mass (Table 2), with a Ca/P ratio varying between 1.9 and 2.2. Albeit slightly lower, these values are similar to those reported by Margalida et al.^2^ in fecal samples taken from Pyrenees bearded vultures, and also point to the existence of calcium phosphates in modern scats. The Ca/P ratio for amorphous calcium carbonate ranges between 1.2 and 2.2, while for hydroxyapatite it is 1.67^34^. Modern scat Ca/P ratios (Table 2) are within the Ca/P interval for amorphous calcium carbonate. Nevertheless, the later are transient calcium carbonate phases that can later recrystallize as hydroxyapatite^34^. The presence of Ca is also associated with calcite and vaterite, identified by XRD.

**Table 2:** Calcium (Ca) and phosphorus (P) contents measured in modern bearded vulture scats and the Ca/P ratio.

Sample ID#	Calcium (Ca) %	Phosphorus (P) %	Ca/P
#5	26.3 ± 0.3	12.2 ± 0.2	2.2
#6	26.9 ± 0.2	12.5 ± 0.1	2.2
#7	25.8 ± 0.1	13.4 ± 0.0	1.9
#8	25.2 ± 0.4	11.4 ± 0.0	2.2
#9	24.6 ± 0.5	12.2 ± 0.3	2.0

## Discussion

### Coprogenic inferences

Identifying the biological agents involved in faunal accumulation and modification is critical for expanding our understanding of Palaeolithic subsistence and economy^36–39^. Taphonomic criteria and diagnostic features are provided primarily by actualistic studies and, in this regard, coprogenic stocks are no exception. Here, the comparison of the ALV coprolites and modern bearded vulture scats has been essential for the identification of this trace maker at the Lagar Velho site. To date, the fossil faeces of this raptor recovered at archaeological sites have not been described, with the exception of coprolites found at the Noisetier cave, where several elements in the site’s sedimentary component were identified as bearded vulture coprolites, although they were shapeless fragments and not described in any detail^16^.

Pleistocene coprolites are usually attributed to different morphotypes based on their distinctive criteria^22,24^. To date, three morphotypes have been proposed for the identification of coprolites and their biological agents: (1) morphotype 1—globular, crumbly texture, hard consistency and scarce bone inclusions; (2) morphotype 2—cylindrical shape, spiral internal fabric, friable, abundant bone contents, and (3) a supposed morphotype 3—larger size, crumblier texture and strong hardness. Morphotype 1 corresponds to hyenid coprolites. Due to their hard consistency and distinctive shape they are easily identified and recovered^21,40^. Morphotype 2 corresponds to non-hyenid carnivores, such as wolf, fox, lynx, among other similar predators^23,24,29,30^. Finally, the putative morphotype 3 appears to be related to larger carnivores.

**Figure 8:**
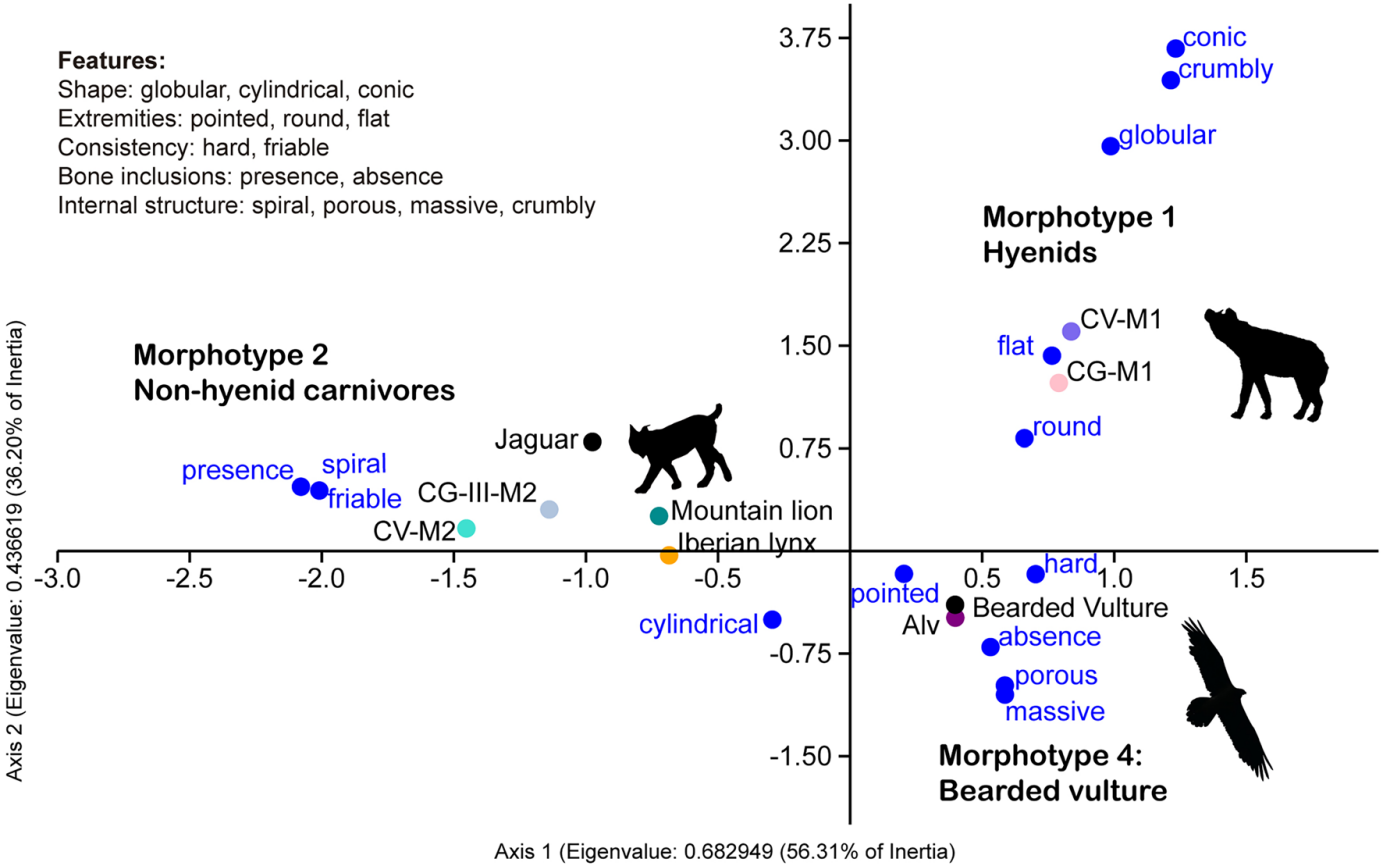
Correspondence analysis of the features (in blue) described for coprolites (ALV, CV: Cova del Coll Verdaguer and CG: Cova del Gegant) and modern-day scats (jaguar, mountain lion, Iberian lynx, bearded vulture). Source data from present study and previous work^22,23,30–32^.

Here, because of the specific characteristics observed in the ALV coprogenic samples, we propose the creation of a new morphotype—morphotype 4—based on a set of attributes that allow the identification of bearded vulture coprolites (Table 3). The internal texture of these coprolites is homogeneous, in general porous, but also massive. Together with the absence of bone inclusions, these features are exclusive to this morphotype (Fig. 8). Other features presented by the bearded vulture coprolites are shared with the other morphotypes, including a cylindrical shape (morphotype 2), a hard consistency (morphotype 1), and the shape of the extremities (morphotypes 1 and 2). Morphology in combination with metrics constitutes a valuable tool for identifying trace markers in archaeological and natural assemblages. Here, the diameter measurements of both bearded vulture coprolites and scats were critical for discerning agency. Although measurements overlap with those of some small carnivores they differ quite markedly from those of the most commonly found coprolites (Fig. 6), making this measure a valuable complement to qualitative data. If an overlap is observed with other small carnivores, such as mustelids or canids, other arguments such as the morphological description and contents might be essential for discerning trace markers. For example, bones are common in many mammalian scats, including small carnivores such as martens or foxes^41,42^; in contrast, they are not found in bearded vulture scats and coprolites. The presence of digested and partially digested bones in the archaeological record of layer 143 is attributed to this avian scavenger and support the bearded vulture as the coprogenic agent. Finally, due to rock-shelter sedimentation and alteration processes, ALV coprolites present various colours—from a yellowish-brown to whitish-grey—while modern-day bearded vulture scats are mainly greyish-white in colour because they have not been buried.

**Table 3:** Morphotype 1 and morphotype 2 coprolite features, according to Sanz^22^, and the new morphotype (4) proposed here, exclusive to the bearded vulture.

	Morphotype 1	Morphotype 2	Morphotype 4
Shape	1. In general globular, although several pellet shape types are identified	1. Cylindrical	1. Cylindrical
Extremities	2. Rounded	2. Sharp-pointed	2. Both sharp-pointed and rounded
Texture	3. Crumbly texture with thick aggregates	3. Spiral distribution with no aggregates	3. Homogeneous. Mostly porous, but also massive
Consistency	4. Hard	4. Friable	4. Hard
Bone inclusions	5. Scarce. Small fragments of cancellous and compact tissues	5. Abundant. Leporids and small mammals. Large mammals scarce	5. No bones
	6. Few and unidentifiable bones	6. Many bones and usually identifiable	

Morphotype 3 has not been included as it still requires validation.

The ingestion of large amounts of bone and the gastrointestinal system of the bearded vulture, capable of dissolving bones, is evident in their faeces. As expected, the main inorganic component of bones—that is, calcium phosphate compounds—are detected in ALV coprolites and in bearded vulture scats. The highly acidic stomach (pH less than 1) of the bearded vulture is capable of disintegrating bones within 24 hours^18^. Accordingly, no bones are found in its faeces. In contrast, small animal bones or fragments of bone are often found in hyenid coprolites^22^. Although it has been widely proposed that hyenids have a very acidic digestive tract, no data are available on the pH of the hyenid stomach to date^43^. This suggests that the presence of bone residuals in faeces and coprolites may be related to other mechanical, chemical or physical factors, as well as to an acidic pH. However, the total absence of bone content in bearded vulture faecal material indicates that these raptors are able to dissolve all bones much more effectively than hyenas.

Finally, coprolites are a valuable source of palaeoenvironment data, because the pollen trapped inside them are well preserved, thanks to their resistance to strong acids and basic environments^44^. For this reason, we can confidently conclude that the ALV coprolites recently analysed for palynology^45^ can be attributed to the bearded vulture. Indeed, these coprolite specimens present an excellent degree of pollen preservation originating from a great diversity of plant species (including *Pinus*, Poaceae, *Erica*, *Artemisia*, *Juniperus* and *Quercus*). The palynological study pointed to a semi-forested landscape, characterised by a rich diversity of tree and shrub formations surrounding the site, dated to c. 29 ka cal BP. Based on the outcome of this study, it is evident that bearded vulture coprolites have a high potential as a source of pollen. However, one issue that requires further research here concerns the possible bias in the pollen record attributable to the behaviour of this avian species.

### Past bearded vulture occupancies

**Figure 9:**
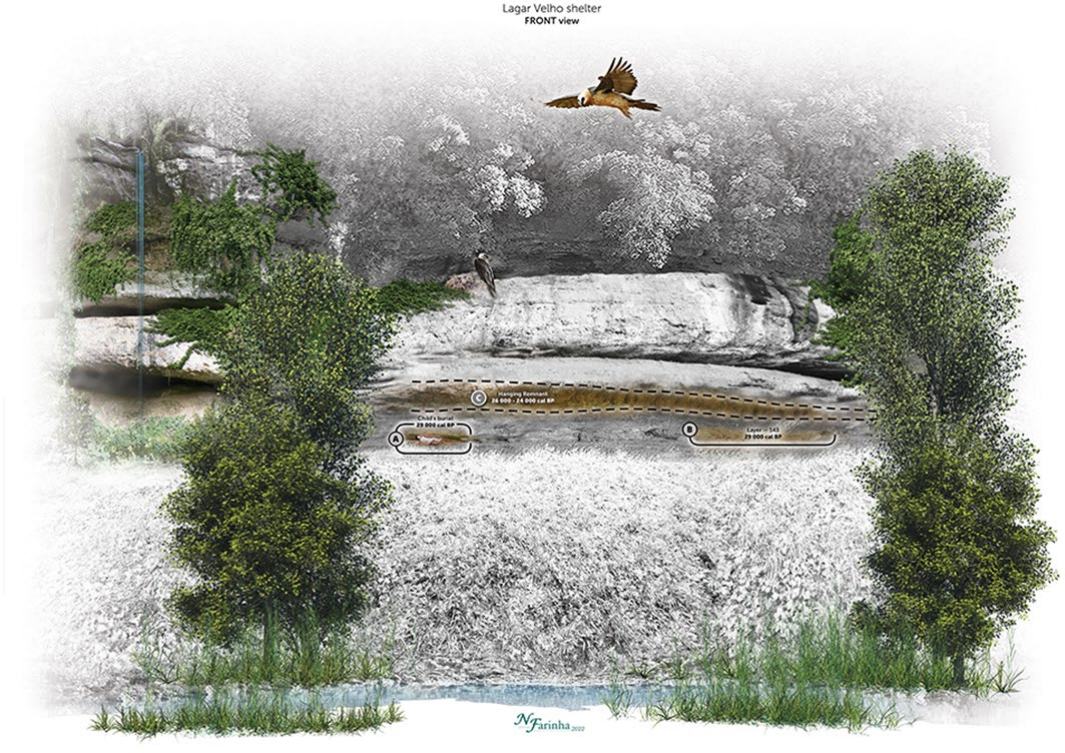
Illustration of Lagar Velho rock shelter viewed from north recreating a bearded vulture couple nesting in a recess above the occupation area, 29 ka cal BP. (**A)**—location of the infant burial; (**B**)—layer 143 from where the studied coprolites were recovered; (**C**)—the Hanging Remnant, a recess preserving sediments with Upper Palaeolithic remains dated from 26 to 24 ka cal BP^26^. Nuno Farinha (adapted from a previous version published in Leiria Museum catalogue).

When feeding their chicks, bearded vultures carry flesh to their nests, mainly food with high meat content^46^. Nests are typically built in well-protected caves or rock shelters, or on the overhangs of steep, vertical relief features^47^. The accumulation of bones in the nest is primarily for adult feeding, but (1) some will not be consumed; (2) others will be partially digested and expelled, with clear evidence of digestive damage, and (3) others will be digested and eventually defecated. The identification of bearded vulture coprolites, together with regurgitated bones, suggests that this raptor used the escarpment recesses above ALV for nesting. In fact, the Lagar Velho rock shelter is located at the base of a limestone cliff, on the south side of the Lapedo Valley characterized by deep vertical walls and horizontal crevices and ledges that would surely have been inaccessible by foot. It is plausible that the bearded vulture nested on these overhang shelves, some located 5 to 20 m above the ground and the archaeological site. Today some of these shelves are filled with sediments and others are exposed rocks with no current evidence of vulture nesting, including griffon vultures. Today, the bearded vulture is extinct in Portugal^48^; however, historically, it was distributed throughout the southern Palaearctic. Various factors, including food availability, animal husbandry and hunting, led to a decline in population decline and to regional extinctions over the last two centuries^47^. Thus, its current distribution in the Iberian Peninsula is limited to just a few areas, such as the Pyrenees, but before its population declined, the bearded vulture was common in most of the Iberian mountain systems^6,49^. As our coprolite specimens demonstrate, the outcrop above the ALV site may have lodged a nest-site of bearded vultures (Fig. 9).

Studies have shown that human activities can affect the breeding and reproductive success of various species, including the bearded vulture^50^. Human disturbance reduces the nest attendance of these raptors, making the coexistence of human occupancies and bearded vulture nesting at the same site in the past implausible. At El Mirón (Spain), the bearded vulture used the cave intermittently for nesting over a period of some 5000 years, while human occupancies were limited to the summer months, by when fledging vultures would have abandoned the nest^13^. As such, humans and raptors alternated seasonally the use of the cave and never shared the same space simultaneously.

The bearded vulture is a scavenger that collects bones from medium ungulates killed by carnivores or humans, or strips them from the carcasses of animals that have died from natural causes. The bone-based diet of the bearded vulture certainly has advantages over that of meat-eating scavengers because while the meat and soft tissues of a carcass decay quickly, bones and marrow are edible for longer periods: dry bones, for example, retain 90% of the protein found in fresh bones^5^. This means the bearded vulture can live in areas of low ungulate biomass, such as Tibet or the Himalayas, thanks to the longer-term benefit of carcasses^18^. The zooarchaeological results obtained at El Mirón suggest that the bearded vulture transported bones into the cave and did not scavenge the food remains discarded by hunter-gatherer groups^13^. The bearded vulture is also considered to be the primary bone accumulating agent in the Noisetier cave^16^ based on mutually exclusive prey selection: i.e. vultures targeted chamois and ibex carcasses while humans focused on hunting red deer.

Faunal remains found in Palaeolithic assemblages are extremely fragmented due to butchery practices, marrow recovery^17,51^ and human and animal trampling, strengthening the hypothesis that bearded vultures do not scavenge on anthropic accumulations. Nevertheless, other hypotheses need to be considered to explain the presence, behaviour and footprint of this vulture in archaeological sites. Moreover, the range of human activities^52,53^ undertaken at these locations is extremely varied in terms of purpose, duration, rhythm and the way the space was occupied. All these factors have to be taken into account when interpreting Palaeolithic sites and the interactions that were established between humans and other species, such as the bearded vulture. At ALV, the interactions between humans and the bearded vulture are still under investigation and further data are required before any interpretation can be confirmed.

## Conclusion

Coprolite studies are essential for evaluating the involvement of different biological agents in the formation of an archaeological assemblage. They are also useful when seeking to obtain a more comprehensive picture of the dynamics established between humans and predators at a given site. The identification of bearded vulture coprolites at Lagar Velho rock shelter complements previous investigations and stresses the importance of coprolites for the identification of this avian in archaeological and palaeontological sites. The distinctive features observed in the modern-day bearded vulture and in the coprogenic samples from ALV enable us to establish a new morphotype, in addition to the three already proposed, which we have named morphotype 4. Among the criteria proposed for its identification are its cylindrical shape; diameter; pointed extremities; its homogeneous, porous, but also massive, internal texture; its hard consistency and the total absence of bone inclusions. In short, bearded vulture coprolites present diagnostic attributes that can be readily identifiable in faunal assemblages, and which enable us to detect the activity of this frequently unnoticed agent in archaeological sites. The presence of this avian scavenger at ALV suggests the use of the rock shelter escarpment as a nesting site. Ongoing research at Lagar Velho is sure to shed more light on the past behaviour of Upper Palaeolithic hunter-gatherers and bearded vultures and their interactions.

## Materials and methodology

### Coprolite and bearded vulture scat analyses

The coprogenic assemblage analysed here originates from fieldwork undertaken at ALV in 2018. A total of 76 coprolites recovered from layer 143 of squares H4 to H7, G5 and G6 of the excavation grid were selected for this study, based on their state of preservation.

Modern-day bearded vulture faeces were collected by several collaborators at the Ordesa and Monte Perdido National Park. Bearded vulture scats are easily distinguishable from other vulture droppings also present at the feeding stations. Unlike those of bearded vultures and mammals, griffon and other vulture droppings are whitish splodges containing a paste of urinary and faecal waste and are easy to differentiate. Several biologists and APNs (Agentes de Protección de la Naturaleza) collected the samples from nests and from the feeding stations located in the aforementioned National Park as well as in other areas of Aragon (Spain). We withhold the precise locations of the nests in order to protect the species. Additionally, several scats from bearded vulture chicks (aged between 40 to 100 days) kept at the Breeding Centre for the Lammergeier in Human Isolation (CRIAH) (Zaragoza, Spain) were provided by the *Fundación para la Conservación del Quebrantahuesos* (FCQ). These chicks were raised from eggs removed from nests in the Aragonese region. All samples were placed individually in zipper plastic bags. A total of 81 specimens were selected for the present study.

The ALV coprolites and the modern scats were analysed macroscopically by adapting the standardized method proposed by Jouy-Avantin^54^ and Sanz^22^. The analyses focused on the following attributes (and so all shapeless specimens were discarded at the outset from these analyses): (1) preservation status (complete or fragmentary); (2) morphology (cylindrical or tube-like, spherical or globular, conical, tubercle-like and undetermined); (3) shape of extremities (round, sharp-pointed, pointed or flat/concave); (4) internal texture (crumbly, porous, crumblier, internal spiral fabric and undetermined), including hardness (friable or hard) and constriction lines; (5) morphometrics (maximum length and diameter in well-preserved specimens) and (6) bone content. For bone content analyses, the outer (external) surface and the sections of fragmented coprolites were examined to identify bone and tooth inclusions. Coprolites and scats were not disaggregated in order to identify these items. The results were compared with measurements reported in earlier studies of both fossil and modern scats^22,23,30,33,55^. As actualistic and coprogenic studies show the diameter to be the most important metric for distinguishing carnivore agents, this measure was duly employed in the present study. PAST 4.06b software^56^ was used to compare the diameter measurements and characteristics of the ALV coprolites and bearded vulture faeces with other taxa scats.

### Mineralogical and elemental composition of scats and coprolites

The mineralogical and elemental composition of four coprolites and five modern-day bearded vulture scats (Supplementary Table 2) was analysed at the *Centres Científics i Tecnològics de la Universitat de Barcelona* (CCiTUB).

All samples were analysed using X-ray diffractometry (XRD) to determine their mineralogy. The peaks in the resulting diffractograms reflect the different planes formed by the atoms of each crystallized mineral present in the samples, allowing for its identification. Amorphous materials (including organic matter) do not produce diffraction patterns as they do not have a crystalline structure. For the analyses, a representative quantity of each sample (coprolite/scat) was ground using an agate pestle and mortar. The ground samples were then mounted in cylindrical cavities—diameter of 16 mm diameter and a thickness of 2.5 mm—and placed in standard (PW1811/16) sample holders.

The analyses were performed in a PANalytical X’Pert PRO MPD alpha1 powder diffractometer in Bragg–Brentano θ/2θ geometry of 240 mm of radius using Cu Kα1 radiation (λ = 1.5406 Å) at 45 kV–40 mA, using a focalizing Ge (111) primary monochromator and a PIXcel detector working at an active length of 3.347º. During the analyses, samples were spun at 2 revolutions s^−1^. A variable automatic divergence slit, to ensure an illuminated length in the beam direction of 10 mm, a mask defining the length of the beam over the sample in the axial direction of 12 mm and Soller slits limiting the diffracted beam to 0.04 radians, were used.

The measurement range (2θ) was from 4 to 100º with a step size of 0.026º and a measuring time of 100 s. Four consecutive scans were performed.

Phase identification was performed based on comparisons between the detected reflections and the values displayed in the PDF (*Powder Diffraction File*) database, published by the ICDD-JCPDS (International Centre for Diffraction Data and Joint Committee of Powder Diffraction Standards, 2021).

Elemental analyses were also conducted on the modern scats (samples ID#5 to 9; Supplementary Table 2). Prior to analysis, 0.1 g of each sample was digested with HNO_3_ and H_2_O_2_ in a Teflon reactor heated to 90 ºC in an oven and after diluted with distilled H_2_O to a dilution of 1/20. The final volume was calculated by weight and weight/volume ratio. Quality assurance was performed by measurement of triplicate samples and three blanks.

Determination of calcium (Ca) and phosphorus (P) was performed by inductively coupled plasma optical emission spectroscopy (ICP-OES) in a Perkin Elmer Optima 8300 equipment under standard conditions. Calibration of the equipment was complete using five standard samples prepared from certified standard solutions traceable to NIST.

## Supplementary Information

Supplementary Information 1.

Supplementary Information 2.

Supplementary Information 3.

## Data Availability

All data generated during this study are included in this published article and its supplementary information files.
