# Supplementary material for: The characterization of bearded vulture (Gypaetus barbatus) coprolites in the archaeological record
Source: Sci Rep. 2023 Jan 3;13:57. doi: 10.1038/s41598-022-25288-x (PMC9810590; doi:10.1038/s41598-022-25288-x)

The characterization of bearded vulture (*Gypaetus barbatus*) coprolites in the archaeological record

Montserrat Sanz[1,2*]

Joan Daura[1,2]

Ana Maria Costa[3,4,5,6]

Ana Cristina Araújo[2,3,4]

[1]Grup de Recerca del Quaternari, GRQ-SERP. Department of History and Archaeology, University of Barcelona, 08001, Barcelona, Spain. montsesanzborras@ub.edu, jdaura_lujan@ub.edu

[2] UNIARQ-Centro de Arqueologia da Universidade de Lisboa, Faculdade de Letras, Universidade de Lisboa, 1600-214, Lisboa, Portugal.

[3]Laboratório de Arqueociências (LARC)-DGPC, Calçada do Mirante à Ajuda, nº 10A, 1300-418, Lisboa, Portugal. acosta@dgpc.pt, acaraujo@dgpc.pt;

[4]CIBIO - Centro de Investigação em Biodiversidade e Recursos Genéticos | InBIO Laboratório Associado | BIOPOLIS - Programme in genomics, biodiversity and land planning, Vairão, Portugal

[5]IDL - Instituto Dom Luiz, Universidade de Lisboa, Campo Grande, Edifício C6, Piso 3, 1749-016 Lisboa, Portugal

[6]IIIPC - Instituto Internacional de Investigaciones Prehistóricas de Cantabria, Universidad de Cantabria - Gobierno de Cantabria-Santander, Avda de los Castros 52, 39005 Santander, Spain

*Corresponding author

Supplementary Figure 1. A-B. Maps showing the location of the Lagar Velho rock shelter (red star) in Portugal. Map extracted from OpenStreetMap (CC BY-SA). OpenStreetMap© licensed under ODdL 1.0 (https://www.openstreetmap.org/copyright) by the OpenStreetMap Foundation (OSMF). ©OpenStreetMap contributors (https://www.openstreetmap.org/). The licence terms can be found on the following link: http://creativecommons.org/licenses/by-sa/2.0/ (accessed on 21 May 2022). C. Plan of the site and Excavation area where coprolites were studied (squares marked in blue). D. Excavation area.

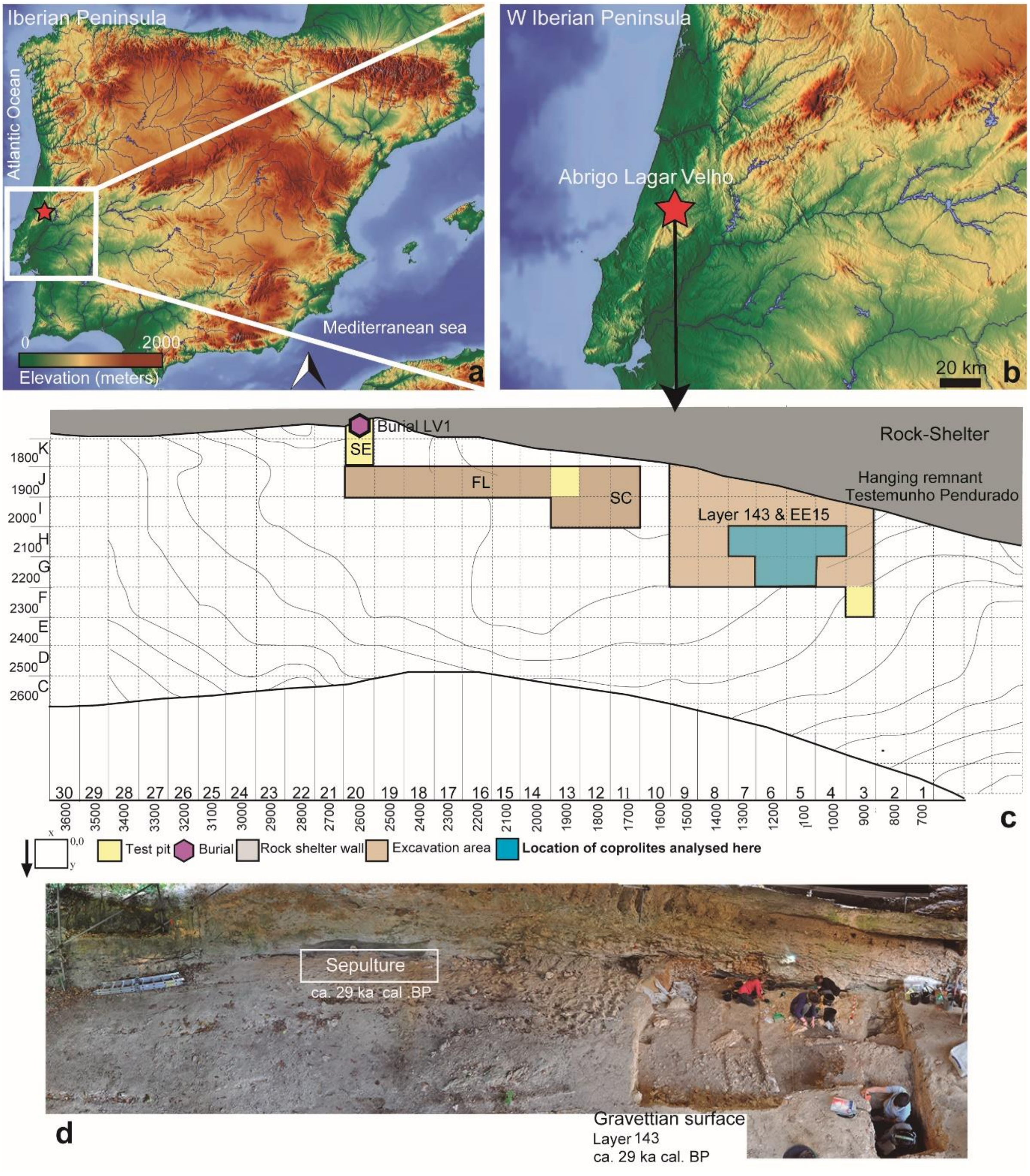

Supplementary Figure 2. Partially (d) and completely (a-c) digested bones from layer 143. Note the rounded fracture edges, the polishing, the slimming of fracture edges and the perforations.

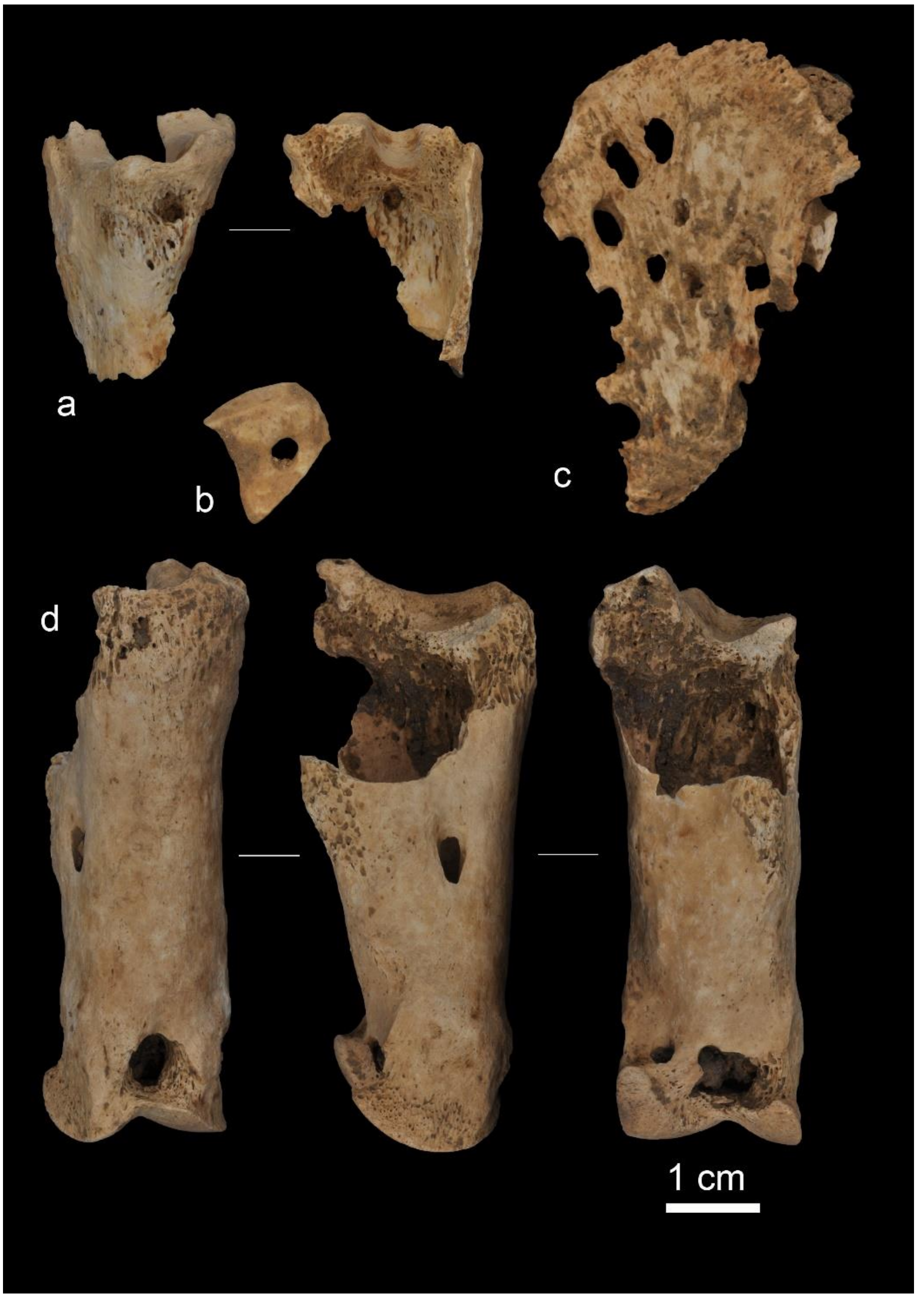

Supplement: Supplementary file 1 — Supplementary Information 1. [file 41598_2022_25288_MOESM1_ESM.pdf]
