# Supplementary material for: The characterization of bearded vulture (Gypaetus barbatus) coprolites in the archaeological record
Source: Sci Rep. 2023 Jan 3;13:57. doi: 10.1038/s41598-022-25288-x (PMC9810590; doi:10.1038/s41598-022-25288-x)
Supplement: Supplementary file 3 — Supplementary Information 3. [file 41598_2022_25288_MOESM3_ESM.pdf]

Supplementary Table 2. Coprolites from ALV (#1 to # 4) and the modern-day scats (#5 to # 9) of bearded vultures analysed.

Sample ID#	Site ID	Provenance	Description	Collection date
#1	1462	ALV-G5-143	Coprolite	2018 field campaign
#2	1521	ALV-G6-143	Coprolite	2018 field campaign
#3	1532	ALV-G6-143	Coprolite	2018 field campaign
#4	1148	ALV-H4-143	Coprolite	2018 field campaign
#5	-	Escuaín (Aragón)	Modern scat	17.11.2020
#6	-	Escuaín (Aragón)	Modern scat	24.11.2020
#7	H3	CRIAH	Modern scat	2020
#8	-	Nest (Aragón)	Modern scat	9.3.2021
#9	P	CRIAH	Modern scat	2020
